# De novo characterization of placental transcriptome in the Eurasian beaver (*Castor fiber* L.)

**DOI:** 10.1007/s10142-019-00663-6

**Published:** 2019-02-18

**Authors:** Aleksandra Lipka, Lukasz Paukszto, Marta Majewska, Jan Pawel Jastrzebski, Grzegorz Panasiewicz, Bozena Szafranska

**Affiliations:** 1 Department of Gynecology and Obstetrics, School of Medicine, Collegium Medicum, University of Warmia and Mazury in Olsztyn, Niepodległości Str 44, 10-045 Olsztyn, Poland; 2 Department of Plant Physiology, Genetics and Biotechnology, Faculty of Biology and Biotechnology, University of Warmia and Mazury in Olsztyn, Oczapowskiego Str 1A, 10-719 Olsztyn, Poland; 3 Department of Human Physiology, School of Medicine, Collegium Medicum, University of Warmia and Mazury in Olsztyn, Warszawska Str 30, 10-082 Olsztyn, Poland; 4 Department of Animal Anatomy and Physiology, Faculty of Biology and Biotechnology, University of Warmia and Mazury in Olsztyn, Oczapowskiego Str 1A, 10-719 Olsztyn, Poland

**Keywords:** Beaver, Pregnancy, RNA-Seq, De novo assembly, Non-model species

## Abstract

Our pioneering data provide the first comprehensive view of placental transcriptome of the beaver during single and multiple gestation. RNA-Seq and a de novo approach allowed global pattern identification of *C. fiber* placental transcriptome. Non-redundant beaver transcriptome comprised 211,802,336 nt of placental transcripts, grouped into 128,459 contigs and clustered into 83,951 unigenes. An Ensembl database search revealed 14,487, 14,994, 15,004, 15,267 and 15,892 non-redundant homologs for *Ictidomys tridecemlineatus*, *Rattus norvegicus*, *Mus musculus*, *Homo sapiens* and *Castor canadensis*, respectively. Due to expression levels, the identified transcripts were divided into two sets: non-redundant and highly expressed (FPKM > 2 in at least three examined samples), analysed simultaneously. Among 17,009 highly expressed transcripts, 12,147 had BLASTx hits. GO annotations (175,882) were found for 4301 transcripts that were assigned to biological process (16,386), cellular component (9149) and molecular function (8338) categories; 666 unigenes were also classified into 122 KEGG pathways. Comprehensive analyses were performed for 411 and 3078 highly expressed transcripts annotated with a list of processes linked to ‘placenta’ (31 GO terms) or ‘embryo’ (324 GO terms), respectively. Among transcripts with entire CDS annotation, 281 (placenta) and 34 (embryo) alternative splicing events were identified. A total of 8499 putative SNVs (~ 6.2 SNV/transcript and 1.7 SNV/1 kb) were predicted with 0.1 minimum frequency and maximum variant quality (*p* value 10e^−9^). Our results provide a broad-based characterization of the global expression pattern of the beaver placental transcriptome. Enhancement of transcriptomic resources for *C. fiber* should improve understanding of crucial pathways relevant to proper placenta development and successful reproduction.

**Electronic supplementary material:**

The online version of this article (10.1007/s10142-019-00663-6) contains supplementary material, which is available to authorized users.

## Introduction

Within the Rodentia order, the Castoridae family is represented by only two still extant species, *Castor canadensis* in North America and *Castor fiber* in Eurasia. Both species are the world’s second largest rodents after the capybara, and can be distinguished only by cytogenetic analyses (Lavrov and Orlov 1973). Molecular data strongly support the placement of *Castor* genus within a ‘mouse-related clade’, including various families: Pedetidae, Anomaluridae, Muridae, Dipodidae, Geomyidae and Heteromyidae and suggest Geomyoidea to be the closest relatives of the beavers (Montgelard et al. 2008; Blanga-Kanfi et al. 2009). Mitochondrial DNA analyses revealed Anomalurus to be the most closely related to the beavers (Horn et al. 2011).

The *C. fiber* was widespread in Europe and Asia. However, at the beginning of the twentieth century, over-hunting had drastically reduced the population and range of this species. Ongoing conservation programs (management and reintroductions) prevented the beaver population from declining again and, as a result, the beaver species was classified into the Least Concern category by the International Union for Conservation of Nature (http://www.iucnredlist.org/details/4007/0). Despite the success of conservation programs, the *C. fiber* population displays a low level of genetic divergence, at the intra- and interpopulation level (Ducroz et al. 2005). This is characteristic for some species (e.g. the brown bear) that have undergone a severe population bottleneck (Taberlet and Bouvet 1994). To avoid further genetic erosion, responsible and effective management should include a careful selection of the founding stock for reintroduction of endangered species (Senn et al. 2014).

The mammalian placenta is the functional connection between mother and embryo/foetus. Among eutherians (Garratt et al. 2013; Furukawa et al. 2014), there are major differences in placental shape (diffuse, cotyledonary, zonary, discoidal, bidiscoidal), interface type (epitheliochorial, endotheliochorial, hemochorial) and the nature of foeto-maternal interdigitations (folded, lamellar, villous, trabecular, labyrinthine).

The Castoridae placenta has been classified as discoidal shape with hemochorial interface (Willey 1914; Fischer 1971; Mossman 1987, 1991). A fully developed rodent placenta is composed of three major layers: the outer maternal layer (decidual cells and uterine vasculature); a middle region (embryo-originated trophoblast cells); and an inner layer (branched trophectodermal villi) for efficient nutrient exchange (Rossant and Cross 2001; Watson and Cross 2005). The placenta is a key component required for pregnancy maintenance. Characterizing genes crucial for placental development can serve as a basis for identifying mechanisms underlying effective reproduction (Majewska et al. 2017). Detailing the beaver placental transcriptome may provide a valuable genetic resource required for founding stock selection. Therefore, full-length cDNA sequences are extremely essential for determination of gene structures, especially useful for beaver population genotyping.

Next generation sequencing methods, such as RNA-Seq, have enabled the examination of the transcriptome landscape in a range of species without a sequenced genome. Highly efficient and precise annotation tools using reference genomes from related species are evolving rapidly (Ockendon et al. 2016). In case of limited sequence information in public databases, RNA-Seq makes the large-scale discovery of novel transcripts possible (Hong et al. 2014). Therefore, only a de novo approach may be efficient for identification of placental spatio-temporal gene expression in the Eurasian beaver. Only a few studies have been performed with some beaver tissues (Bogacka et al. 2017; Chojnowska et al. 2017; Czerwinska et al. 2017; Lipka et al. 2017a, b; Lok et al. 2017), but none of them concern de novo characterization of the beaver placental transcriptome. Thus, the objective of this study was to identify and characterize the placental transcriptome of the *C. fiber*.

## Materials and methods

### Ethics statement

Eurasian beavers for the study were captured and sacrificed with the consent of the Regional Directorate for Environmental Protection in Olsztyn (RDOŚ-28-OOP-6631-0007-638/09/10/pj). The experimental protocol was also approved by the Local Ethical Commission for Experiments on Animals at the University of Warmia and Mazury in Olsztyn (UWM/111/2011/DTN) and the III Local Ethical Commission for Experiments on Animals at Warsaw University of Life Sciences (11/2010). All possible efforts were made to minimize animal suffering.

### Animals and tissue collection

Discoid placentas (harvested separately from each conceptus) were collected from three beaver females during single, twin and triple late pregnancy (Table 1). Placental tissues were immediately preserved in liquid nitrogen, transported to the Laboratory of the Department of Animal Physiology and stored at − 70 °C until further analyses.

**Table 1:** The overall quality statistics of RNA-seq data for each beaver cDNA library, due to single or multiple gestation type and foetal sex

Gestation type	Cf foetal sex^a^ and sample id	Raw reads	Reads after trimming (Phred > 20)	Number of realigned reads	Number of unaligned reads
Single	F#25	77,677,402	72,128,008	63,887,360	8,240,648
Twin	M#29/1	52,635,236	48,013,138	40,602,464	7,410,674
	F#29/2	68,857,676	63,595,580	54,893,028	8,702,552
Triple	F#26/1	47,720,830	43,492,294	38,123,376	5,368,918
	M#26/2	82,963,544	77,312,070	68,327,446	8,984,624
	F#26/3	54,079,126	49,742,426	43,565,130	6,177,296
Total	6	383,933,814	354,283,516	309,398,804	44,884,712

^a^F, female; M, male

### RNA isolation and Illumina sequencing

Total RNA was isolated from placental tissues using a RNeasy Kit in conjunction with the RNase-Free DNase Set (Qiagen, Germany) as it was described previously (Lipka et al. 2017a). The concentration and purity of total RNA were measured (InfiniteM200; Tecan Group AG, Switzerland) and the RIN values were evaluated (Bioanalyzer 2100; Agilent Technologies, USA). Total RNA samples with high quality (RIN ≥ 8) were subjected to RNA-Seq (OpenExome, Poland). Each cDNA library (Table 1) was constructed accordingly to the manufacturer’s recommendation (TruSeq Stranded mRNA LT Sample Prep Kit, Illumina, USA). Briefly, the main steps of each cDNA library preparation were mRNA capture with oligo dT beads, fragmentation, first and second strand synthesis, end repair, index adapter ligation, adapter fill-in, size selection, indexing PCR and finally quantification. The indexed libraries were diluted and pooled in equimolar ratios, then pair-end sequenced (101 cycles for read 1; 7 cycles for the index read; and 101 cycles for read 2), and 2 × 100 bp reads were obtained (HiSeq2500, Illumina, USA).

### De novo transcriptome assembly

Sequencing quality was evaluated using FastQC software (www.bioinformatics.babraham.ac.uk). Trimmomatic tool (Bolger et al. 2014) was utilized to trim out adaptors and polyA stretches from the raw data, and shorter reads than 50 bp or Phred < 20 were removed from the dataset. Trimmed sequences were de novo assembled with Trinity (Grabherr et al. 2011) software (ver. r20140717; https://github.com/trinityrnaseq/trinityrnaseq/wiki) with a k-mer of 25 on the server (23 cores/120 GB RAM) of the Regional IT Centre (Olsztyn, Poland). Reads from all six samples were combined and the minimum sequence length in the assembly was set to 500 nt.

To create a *C. fiber* ‘reference placental transcriptome’, the obtained transcripts (full-length and alternatively spliced) were assembled (Inchworm, Trinity), then divided into clusters and, for each cluster, a de Bruijn graph was constructed (Chrysalis, Trinity) and paralogs were reported (Butterfly, Trinity). The reference transcriptome was formed as cluster structure with unigene as basic unit. Each unigene was a group of related transcripts included in the same de Bruijn graph (Grabherr et al. 2011), whereas each cluster regarded a single transcript or non-coding sequence.

### Comprehensive analyses of the *C. fiber* placental transcriptome

The Benchmarking Universal Single-Copy Orthologs (BUSCO v1.1) was used to estimate the accuracy of the transcriptome assembly (Simão et al. 2015), based on the percentage of unigenes assigned as putative core vertebrate genes.

Trinitystats.pl script (Grabherr et al. 2011) was utilized to perform overall statistics, including the following parameters: average contig length, GC content, total assembled bases and N50 value. To decrease redundancy, the CD-HIT-EST v4.6 tool was used (Fu et al. 2012), and we removed duplicated transcripts from the reference transcriptome by clustering at the 97% sequence identity. Further, unigenes were searched (BLASTx, cut-off *E*-value 1e^−20^) against four protein ENSEMBL databases (http://www.ensembl.org/) of *Mus musculus*, *Rattus norvegicus*, *Ictidomys tridecemlineatus* and *Homo sapiens* and NCBI protein database *of Castor canadensis*. To check the quality of the assembly, the raw paired-end reads of each sample were aligned back onto the unigenes using Bowtie (Langmead et al. 2009). Read counts for non-redundant sequences were calculated by RSEM software (Li and Dewey 2011) and final expression values were estimated as trimmed mean of *M*-value (TMM)-normalized fragments per kilobase of transcript per million mapped reads (FPKM). The obtained data were divided into two datasets: non-redundant contigs and highly expressed transcripts with FPKM > 2 (Webb et al. 2015) in at least three samples. Both datasets were analysed with Annocript software (Musacchia et al. 2015), in order to identify and characterize transcripts encoding polypeptide precursors. To identify homologous transcripts, the most accurate multiple tools (BLASTx, BLASTn, rpstBLASTn, Portrait, dna2pep) were used to search UniProt, TrEMBL, SwissProt, Pfam and Rfam databases for all available species. Transcripts that showed no coding potential were annotated as possible long non-coding RNA (lncRNA). To confirm transcript as lncRNA by Annocript, it had to meet the following requirements: no match in public databases; a Portrait non-coding probability (> 0.95); transcript length (> 200 nt); ORF (< 300 nt) and coding potential calculator (CPC < 0; http://cpc.cbi.pku.edu.cn/).

Further, highly expressed contigs (FPKM > 2) were aligned to the Rodentia protein NCBI database using CloudBlast (BLASTx-fast) implemented in BLAST2GO (Conesa et al. 2005). Due to the utilized pipeline, transcripts were assigned to three main Gene Ontology (GO) categories: biological process (BP), cellular component (CC) and molecular function (MF). Additionally, each highly expressed transcript (FPKM > 2) was checked for molecular interactions and networks (KEGG pathway; http://www.genome.jp/kegg/pathway.html), conserved domains and final assignment to protein families was performed with the InterProScan v.5 (Jones et al. 2014).

Thorough analyses were performed for highly expressed transcripts involved in the processes related to embryo or placenta development. The AmiGO (Gene Ontology) database was searched with the query ‘placenta’ or ‘embryo’ to create a list of biological processes for each query. The selected processes were then linked with *Rattus norvegicus* genes using the BioMart tool (http://www.ensembl.org/info/data/biomart/) and these genes were blasted with highly expressed *C. fiber* transcripts. Only transcripts with *E*-value (< 10e^−5^) were annotated using a .gff file (output from Annocript) and AUGUSTUS tool (Stanke and Morgenstern 2005), with Eukaryotic GeneFinding option, implemented in BLAST2GO. Selected transcripts with complete CDS were analysed for alternative splicing events (AS) and single nucleotide variants (SNVs). Transcripts within each cluster were aligned using multi-sequence alignment (MSA), in order to identify splice sites for a unigene. For SNV prediction in the selected transcripts, ‘Find Variations/SNPs’ tool in Geneious software (Biomatters LDT) was used with minimum variant frequency (set as 0.1).

## Results

### Processing the sequencing data and de novo assembly

Six constructed and sequenced cDNA libraries (placental mRNAs originated from single, twin and triple pregnancy) revealed a general overview of the beaver placental transcriptome based on the obtained total 383,933,814 of 100 bp paired-end raw reads (Table 1). All reads were deposited in the Short Read Archive (SRA; http://www.ncbi.nlm.nih.gov/Traces/sra/sra.cgi) of the National Center for Biotechnology Information (NCBI) under the BioProject Accession number PRJNA313146. After TruSeq adapter trimming and removal of low-quality reads, 354,283,516 clean reads were obtained. Specific characteristics of each cDNA library are provided with quality statistics (Table 1).

**Fig. 1:**
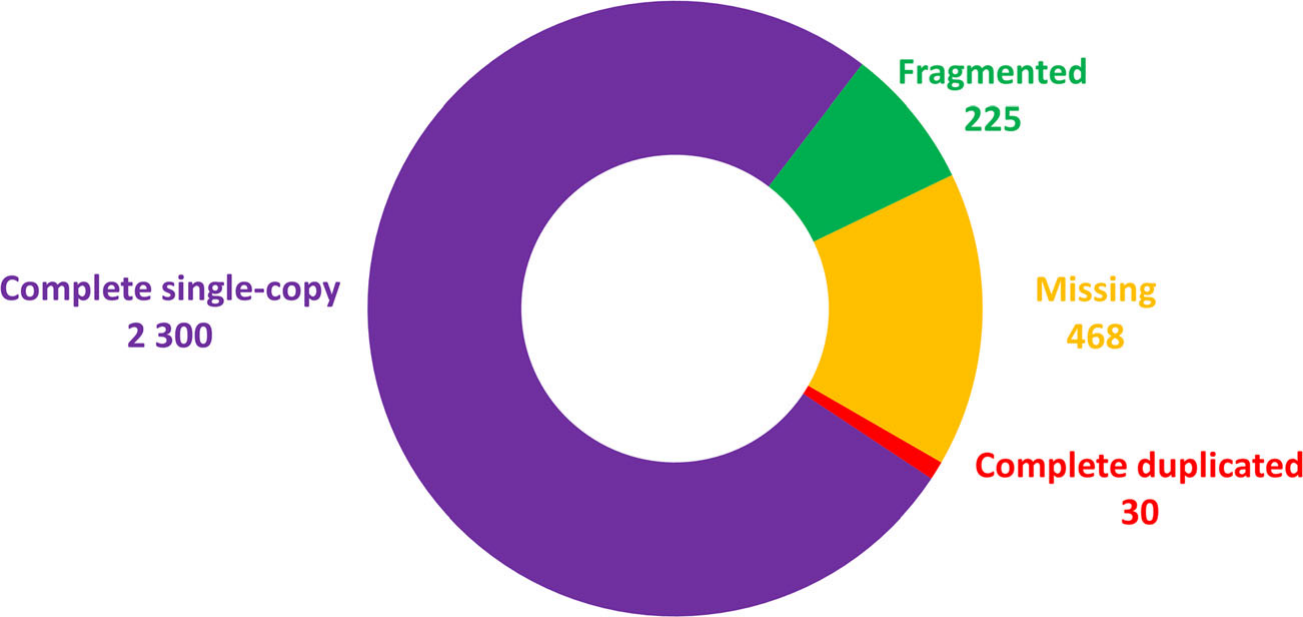
Core genes identified in de novo assembly of the *C. fiber* placental transcriptome. Complete—contig lengths matched to the BUSCO profile; duplicated—contigs found more than once in orthology annotation; fragmented—partially covered; missing—no matches that passed orthology classification tests

**Fig. 2:**
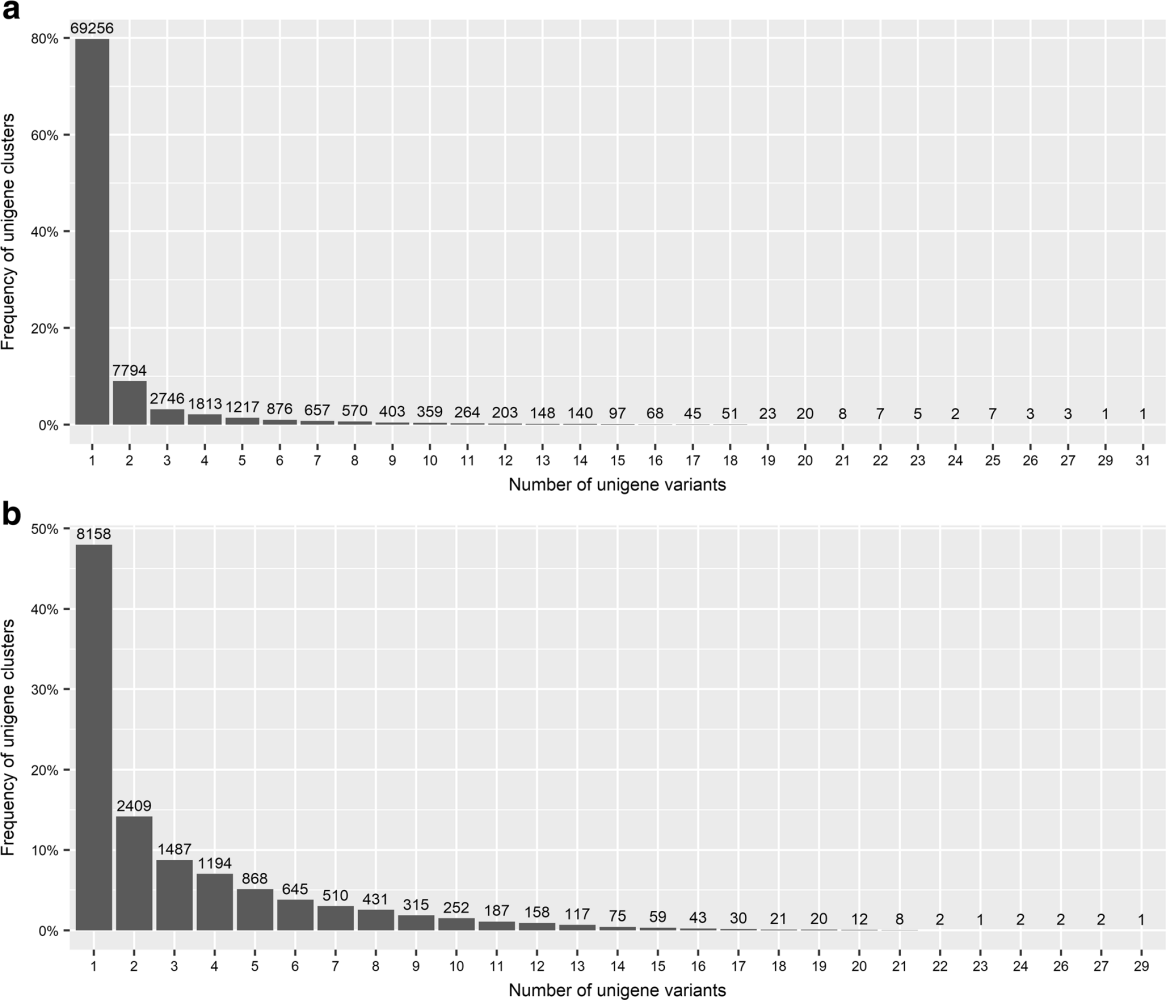
Frequency distribution of contigs: **a** Non-redundant assigned to unique cluster; **b** Assigned to highly expressed (FPKM > 2) unigenes

Reads obtained after trimming (Table 1) were de novo assembled in to the *C. fiber* ‘reference transcriptome’ that comprised of 143,217 contigs (> 500 bp). Among the 3023 core vertebrate genes, BUSCO analyses revealed 77.1% (2330) as complete genes—the sum of single copy (2300) and duplicated (30), 7.4% (225) as fragmented and 15.5% (468) missing transcripts (Fig. 1). More than 84% of the aforementioned core vertebrate genes predicted by BUSCO (complete and fragmented) confirmed the completeness of the transcriptome assembly. Contigs with a sequence similarity > 97% were grouped into 128,459 non-redundant contigs (Table 2) and were afterwards clustered into 83,951 unigenes (Fig. 2a), among them, 17,009 were highly expressed (Fig. 2b).

**Table 2:** De novo assembly statistics for beaver non-redundant contigs

Number of all assembled contigs	143,217
Number of non-redundant contigs	128,459
Total size of contigs	211,802,336 nt
Longest contig	16,751 nt
Shortest contig	501 nt
Number of contigs > 1K nt	69,114 (53.8%)
Number of scaffolds > 10K nt	212 (0.2%)
Mean contig size	1649 nt
Median contig size	1085 nt
N50 contig length	2354 nt
L50 contig count	27,018
%A	26.40%
%C	22.99%
%G	23.87%
%T	26.73%

**Fig. 3:**
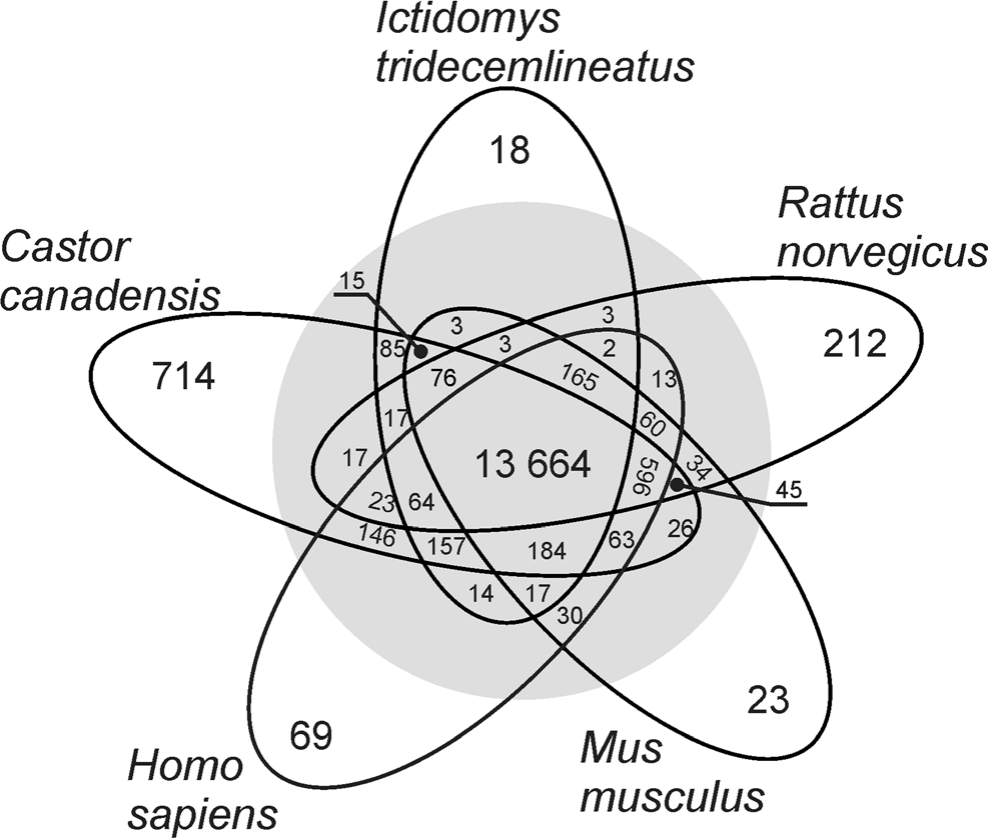
Venn diagram presenting a summary of non-redundant unigene annotations for the beaver placental transcriptome with examined Ensembl and NCBI protein datasets. Overlapping numbers (in the grey circle) indicate frequent BLASTx matches with polypeptide precursors which originated from two or more species. Non-overlapping numbers (out of the grey circle) indicate beaver homologs uniquely blasted with each species

Non-redundant contigs comprised 211,802,336 nt of transcripts with maximum contig length (16,751 nt), N50 (2354 nt) and mean contig length (1649 nt). More than 69K contigs were longer than 1000 nt and 212 contigs were longer than 10,000 nt (Table 2). BLASTx searching of the longest isoform for each beaver unigene with protein databases (cut-off *E*-value 1e^−20^) revealed 14,487, 14,994, 15,004, 15,267 and 15,892 non-redundant homologs for the thirteen-lined ground squirrel (*Ictidomys tridecemlineatus*), the brown rat (*Rattus norvegicus*), the house mouse (*Mus musculus*), the human (*Homo sapiens*) and the Canadian beaver (*Castor canadensis*), respectively (Fig. 3). Among them, 714 *C. fiber* homologs were uniquely identified in the Canadian beaver only, while 212 were identified in the rat, 23 in the mouse and 18 in the squirrel. Also, 69 genes were identified as beaver-specific, not detected in the examined Rodentia species, although existing in human transcriptome (Fig. 3).

**Fig. 4:**
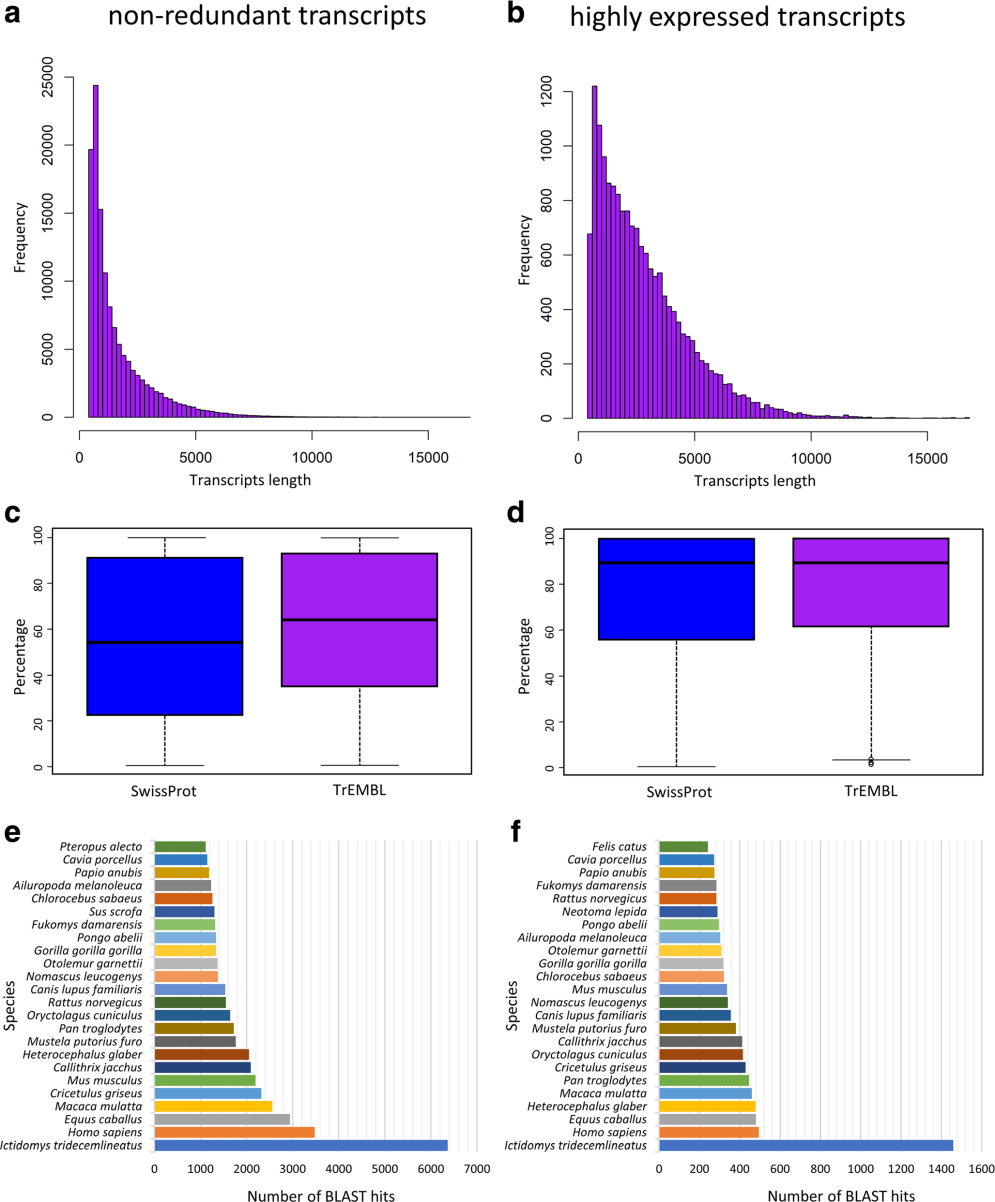
Overall characteristics of de novo identified non-redundant (**a**, **c**, **e**) and highly expressed (**b**, **d**, **f**) transcripts which originated from *C. fiber* placental transcriptome. Frequency distribution of length (**a**, **b**), hit coverage (**c**, **d**) and number of top BLASTx hits by species searches (**e**, **f**) against TrEMBL database

Realignment of the clean reads to the beaver reference transcriptome (MAPQ > 30) revealed 94.9% (#25), 94.3% (#26/1), 94.9% (#26/2), 94.4% (#26/3), 92.9% (#29/1) and 93.7% (#29/2) of mapped reads in each sample, which confirmed successful de novo assembly. Each transcript expression level was estimated based on read counts and TMM-normalized FPKM values (data not shown). Due to expression levels, the obtained data were divided into two data transcript sets: all non-redundant (Fig. 4a, c, e) and highly expressed (FPKM > 2 in at least three investigated samples; Fig. 4b, f, g), analysed simultaneously to fully characterize beaver placental transcriptome.

Such an approach enabled identification of global beaver placental transcriptome as well as a detailed description of highly expressed transcripts, potentially tissue-specific. For non-redundant contigs and highly expressed transcripts, the mean transcript lengths were 1648 nt (Fig. 4a) and 2816 nt (Fig. 4b), respectively. Homology analysis revealed median distribution of hit coverage (within SwissProt and TrEMBL) close to 95% for highly expressed (Fig. 4d), but 60% for non-redundant transcripts (Fig. 4c). The majority of the top BLAST hits of beaver non-redundant/highly expressed unigenes (6365/1459) matched *Ictidomys tridecemlineatus* (Fig. 4e, f), while less to *Homo sapiens* (3475/494), *Equus caballus* (2943/480) and *Macaca mulatta* (2560/460).

### Functional annotation

**Fig. 5:**
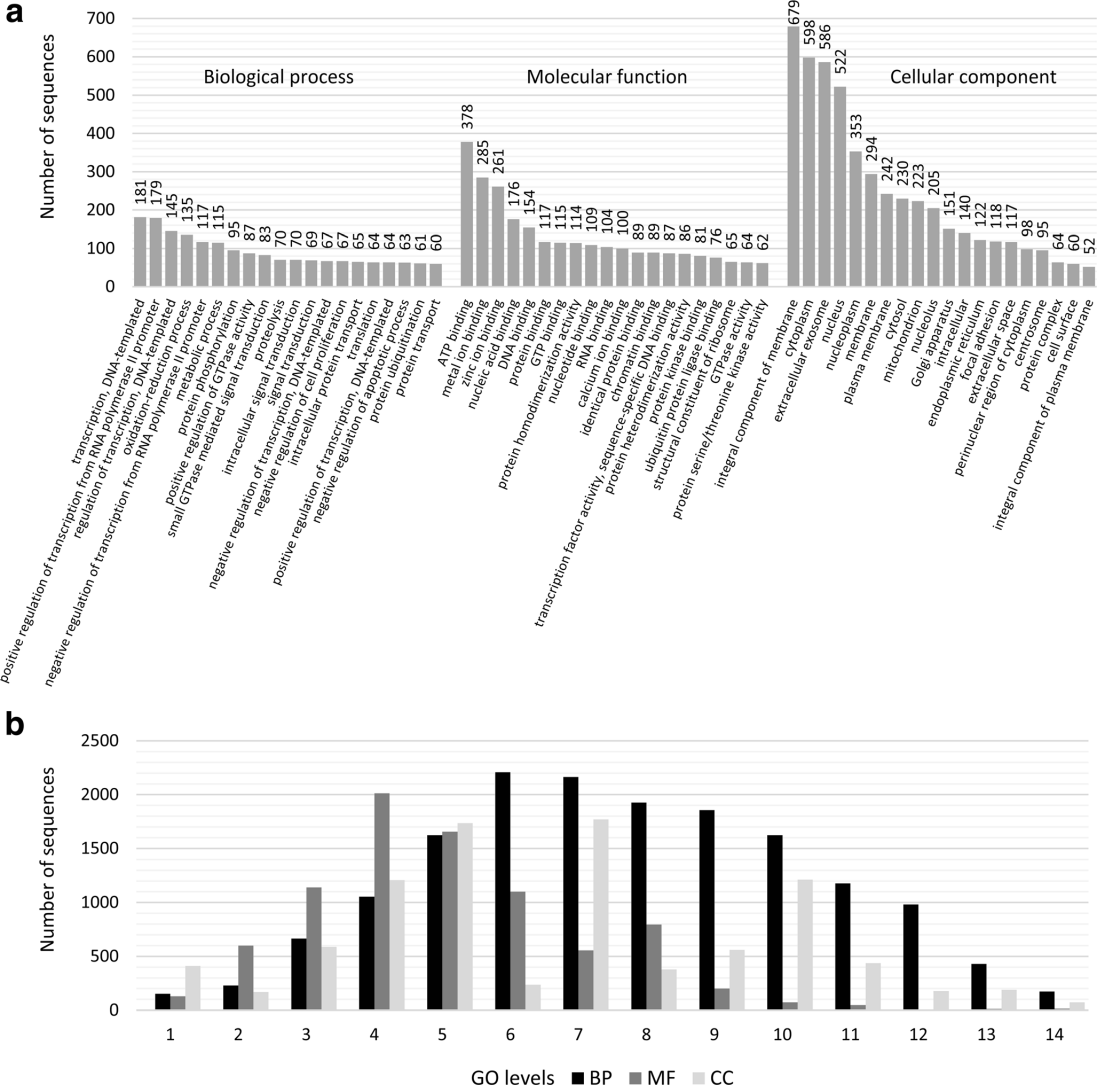
Functional distribution of the highly expressed beaver placental transcripts (**a**) linked to the top 20 biological process—BP, molecular function—MF and cellular component—CC. Classified due to GO level (**b**)

Within 17,009 highly expressed unigenes (FPKM > 2), 12,147 had hits in BLASTx and 1179 transcripts were classified as non-coding (portrait probability > 0.95 and the length of the ORF was shorter than 300 nt). Among the unigenes with BLAST hits, multiple 175,882 GO annotations (Fig. 5a, Table S1) were found for 4301 transcripts and were uniquely assigned to biological process (16,386), molecular function (8338) and cellular component (9149). Functional distribution of the highly expressed beaver placental transcripts classified due to GO level revealed the highest number of annotations within levels 6 and 7 of the BP category (Fig. 5b).

Among the biological process annotations (Fig. 5a), ‘transcription, DNA-templated’ category was overrepresented (181 unigenes), followed by ‘positive regulation of transcription from RNA polymerase II promoter’ (179) and ‘regulation of transcription, DNA-templated’ (145). Due to placental transcriptome changes as the pregnancy advanced, one directly linked biological process category was ‘in utero embryonic development’, represented by 52 unigenes (Table S1). The molecular function category (Fig. 5a) included 3990 unigenes, which were assigned to 1678 GO terms and the majority of unigenes were associated with binding: ATP (378 unigenes), metal ion (285) and zinc ion (261). Within the cellular component category (Fig. 5a), the highest number of transcripts matched to ‘integral component of membrane’ (679 unigenes), ‘cytoplasm’ (598), ‘extracellular exosome’ (586) and ‘nucleus’ (522).

Within the KEGG database, 666 unigenes were classified into 122 pathways that showed 13, 4 and 4 expressed transcripts involved in ‘steroid hormone biosynthesis’, ‘steroid biosynthesis’ and ‘steroid degradation’, respectively (Table S2). Searching of PFAM, SMART, PANTHER and INTERPRO databases revealed structure annotations and membership in the protein family and domain for 3719, 1917, 1127 and 4717 unigenes, respectively. Products of the highly expressed transcripts with the highest number of annotations (top 10 within each of the aforementioned databases) were presented in the Table 3.

**Table 3:** Top 10 annotation hits for products of the highly expressed transcripts within PFAM, SMART, PANTHER and INTERPRO databases

Database	ID	Name	Number of sequences
PFAM	PF00069	Protein kinase domain	82
	PF00076	RNA recognition motif domain	64
	PF00400	WD40 repeat	55
	PF00071	Small GTPase superfamily	55
	PF00096	Zinc finger, C2H2	40
	PF00651	BTB/POZ domain	34
	PF00271	Helicase, C-terminal	32
	PF07714	Serine-threonine/tyrosine-protein kinase catalytic domain	30
	PF12796	Ankyrin repeat-containing domain	29
	PF00169	Pleckstrin homology domain	28
SMART	SM00355	Zinc finger, C2H2-like	103
	SM00220	Protein kinase domain	75
	SM00320	WD40 repeat	72
	SM00360	RNA recognition motif domain	60
	SM00409	Immunoglobulin subtype	50
	SM00184	Zinc finger, RING-type	44
	SM00225	BTB/POZ domain	39
	SM00326	SH3 domain	39
	SM00233	Pleckstrin homology domain	38
	SM00248	Ankyrin repeat	32
PANTHER	PTHR24070	Small GTPase superfamily, Ras type	16
	PTHR23239	Intermediate filament protein	9
	PTHR11937	Actin family	9
	PTHR24322	Short-chain dehydrogenase/reductase SDR	8
	PTHR10516	Peptidyl-prolyl cis-trans isomerase, FKBP-type	8
	PTHR11071	Cyclophilin-type peptidyl-prolyl cis-trans isomerase	8
	PTHR13832	Protein phosphatase 2C family	7
	PTHR10218	Guanine nucleotide-binding protein (G protein), alpha subunit	7
	PTHR11753	Adaptor protein complex, sigma subunit	6
	PTHR18860	14-3-3 protein	6
INTERPRO	IPR027417	P-loop containing nucleoside triphosphate hydrolase	143
	IPR000719	Protein kinase domain	108
	IPR015880	Zinc finger, C2H2-like	103
	IPR007087	Zinc finger, C2H2	102
	IPR001680	WD40 repeat	73
	IPR017986	WD40-repeat-containing domain	69
	IPR000504	RNA recognition motif domain	68
	IPR011009	Protein kinase-like domain	63
	IPR012677	Nucleotide-binding alpha-beta plait domain	61

### Alternative splicing events and single nucleotide variants

**Fig. 6:**
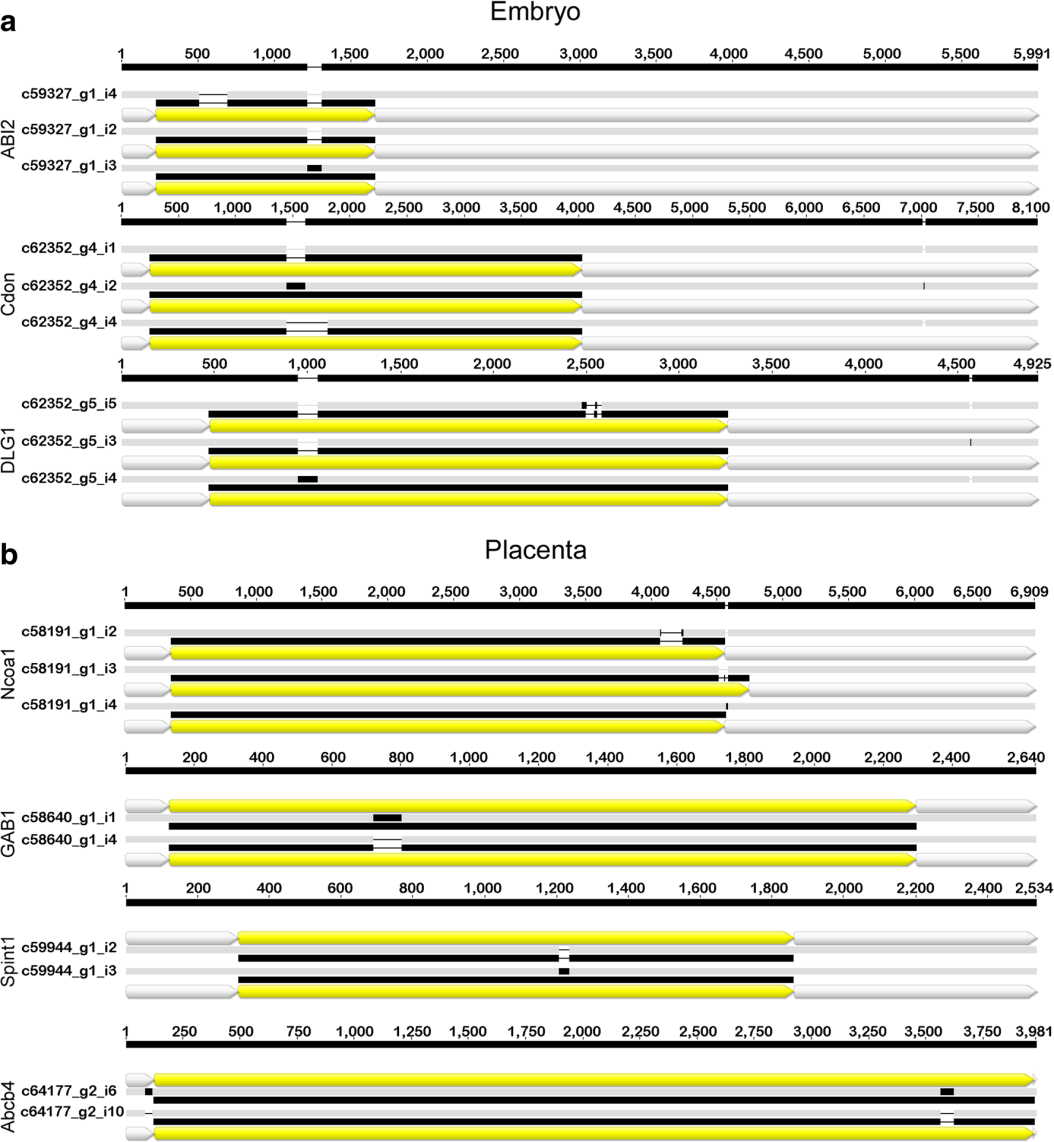
Exemplary multiple and pairwise alignments of putative alternative splicing events in unigenes linked to **a** ‘embryo’ and **b** ‘placenta’ GO terms. The 5′ and 3′ untranslated region (white bar), coding DNA sequence (yellow bar) and polypeptide precursor (black bold line) are indicated. Abbreviations: abl interactor 2 (ABI2); cell adhesion molecule-related/downregulated by oncogenes (Cdon); disks large homolog 1 (DLG1); nuclear receptor coactivator 1 (Ncoa1); GRB2-associated-binding protein 1 isoform X3 (GAB1); Kunitz-type protease inhibitor 1 precursor (Spint 1); phosphatidylcholine translocator ABCB4-like (Abcb4)

Comprehensive analyses of highly expressed transcripts showed that 411 and 3078 contigs were annotated with a list of processes linked to placenta (31 GO terms) or embryo (324 GO terms), respectively. Thereafter, transcripts with Augustus annotation (entire CDS) were selected, which concerned 1244 transcripts associated with embryo and 117 with placenta, and 281 and 34 AS were identified, respectively. Some AS-unigenes were identified with a changed open reading frame (ORF), but without frame shifts, which generated potentially functional polypeptide precursors (Fig. 6).

For the selected placental beaver transcripts with entire CDS (Tables 4 and 5), a total of 8499 putative single nucleotide variants (SNVs) (~ 6.2 SNV/transcript and 1.7 SNV/1 kb) were predicted with 0.1 minimum frequency and maximum variant quality (*p* value > 10e^−9^). Out of these, 611 SNVs were identified within transcripts linked with placenta GO terms (Table 4), including 415 transitions, 186 transversions and 10 multi-nucleotide variants. Predicted 183 SNVs localized in CDS were synonymous (114) and non-synonymous (69). Among transcripts related to embryo GO terms, 7888 SNVs were predicted (Table 5). Of these, 5285 were transitions, less frequent were transversions (2316) and MNVs (287). In CDS regions, 3650 putative SNVs were identified, 1831 changes did not affect amino acid sequence and 1819 were non-synonymous.

**Table 4:** A broad-based characteristic of single nucleotide variants (SNVs) identified in beaver placental transcripts associated with ‘placenta’ GO terms

Gestation	cDNA ID	Polymorphism type		SNVs						MNV	Type of CDS change		Polymorphism localization		All
		Transition	Transversion	A- > C C- > A	A- > G G- > A	A- > T T- > A	C- > G G- > C	C- > T T- > C	G- > T T- > G		Synonymous	Non-synonymous	CDS	UTR	
Single	F#25	75	29	12	36	9	3	39	5	1	25	7	32	73	105
Twin	M#29/1	59	22	6	13	9	5	46	2	–	22	4	26	55	81
	F#29/2	123	58	15	39	13	17	84	13	5	35	40	75	111	186
Triple	F#26/1	50	24	9	25	9	4	25	2	2	7	7	14	62	76
	M#26/2	60	30	14	29	11	3	31	2	2	14	6	20	72	92
	F#26/3	48	23	7	22	9	3	26	4	–	11	5	16	55	71
Total	6	415	186	63	164	60	35	251	28	10	114	69	183	428	611

**Table 5:** A broad-based characteristic of single nucleotide variants (SNVs) identified in beaver placental transcripts associated with ‘embryo’ GO terms

Gestation	cDNA ID	Polymorphism type		SNVs						MNV	Type of CDS change		Polymorphism localization		All
		Transition	Transversion	A- > C C- > A	A- > G G- > A	A- > T T- > A	C- > G G- > C	C- > T T- > C	G- > T T- > G		Synonymous	Non-synonymous	CDS	UTR	
Single	F#25	907	334	101	444	60	76	463	97	29	337	229	566	704	1270
Triple	F#26/1	731	305	77	355	60	82	376	86	30	245	218	463	603	1066
	M#26/2	865	344	100	411	76	75	454	93	46	297	232	529	726	1255
	F#26/3	687	250	64	344	49	63	343	74	28	242	184	426	539	965
Twin	M#29/1	601	242	73	286	53	61	315	55	45	224	167	391	497	888
	F#29/2	1494	841	214	646	200	163	848	264	109	486	789	1275	1169	2444
Total	6	5285	2316	629	2486	498	520	2799	669	287	1831	1819	3650	4238	7888

## Discussion

In this study, RNA-Seq and de novo assembly were conducted for identification of placental transcriptome in the *C. fiber*, which enabled broad-based characteristic of transcripts, including SNVs prediction and functional annotation. RNA-Seq has previously been used to characterize the transcriptomes of some species, however, particularly model organisms with reference genome (Pan et al. 2014; Ropka-Molik et al. 2014). Due to major progress in sequence analysis software, transcriptome studies are now possible for species that do not as yet have a reference genome (Riesgo et al. 2012), of which the beaver is an example. In addition, the availability of the reference genome does not always enable full and effective analyses of the transcriptomic data. For example, domestic duck has a reference genome but it is incomplete, which seriously disrupts the precision of analysis and makes a de novo approach also useful for organisms with a known genome (Zhu et al. 2015).

Our de novo assembly allowed comprehensive characterization of beaver placental transcriptome. A total of 354,283,516 clean reads (Table 1) were generated and assembled into 143,217 contigs of the Eurasian beaver, comprising 211,802,336 Mb of transcriptome (Table 2). The N50 length (2354 nt), average length (1649 nt) of transcripts, the GC content (46.9%) and number of contigs > 1K nt (69,114) indicate successful library construction and good sequencing quality of the *C. fiber* placental transcriptome. Analysis of beaver testes transcriptome permitted to de novo assembly 373 million of high-quality reads into 130,741 unigenes with an average length of 1369.3 nt, N50 value of 1734 and GC content of 46.51% (Bogacka et al. 2017). Our results are very consistent with those obtained in beaver testes, besides the same species was examined, similar methods and parameters were used in both studies that makes the results fully comparable. Experiments performed in reproductive tissues of different species showed that RNA-Seq of Dazu black goat ovary generated 42,377,782 raw reads and 38,771,668 clean reads with the 49.19% GC content, assembled into 150,431 contigs with an average length of 305 nt and N50 length of 479 nt (Zhao et al. 2015). In the swimming crab, de novo transcriptome assembly performed with Trinity using both ovary and testis reads (128,904,126 clean read) generated a total of 80,527 transcripts. The average length of the crab transcripts was 1053 nt and the N50 length was 2439 nt (Meng et al. 2015). Comparison of our data and those obtained in beaver testes (Bogacka et al. 2017) with available results from other RNA-Seq studies indicates that some discrepancies in the statistics of de novo assemblies may be a consequence of the diversity in applied analysis pipelines and examined species. Not without influence remains the spatio-temporal variability and the complexity of each transcriptome, especially in the placenta, an organ that constantly adapts to foeto-maternal interactions. In addition, BUSCO analyses permitted further insight into the completeness and quality of the assembly, which revealed 84.5% of core vertebrate genes in our data. This enabled assembly and annotation completeness to be assessed at a high-quality level. In contrast, the assembly of the Canadian beaver (Lok et al. 2017) and freshwater crayfish (Theissinger et al. 2016) was 70% and 64% complete, respectively. Thus, the de novo assembly obtained in this study was appropriate for the functional annotation and thorough analyses of the Eurasian beaver placental transcriptome.

As the beaver is a non-model species that lacks any prior genome information, searching for closely related organism was conducted. The assembled placental unigenes of the beaver were compared within several databases, which revealed that the majority of the top BLAST hits matched *Castor canadensis* and *Ictidomys tridecemlineatus* (Fig. 3). Also searching of TrEMBL database indicated *I. tridecemlineatus* as closely related to the beaver (Fig. 4e, f)*.* The *Castor* genus is placed within a mouse-related clade including families as Pedetidae, Anomaluridae, Muridae, Dipodidae, Geomyidae and Heteromyidae (Montgelard et al. 2008; Blanga-Kanfi et al. 2009), whereas mitochondrial DNA indicates *Anomalurus* as the most closely related to the beavers (Horn et al. 2011). As the beaver phylogeny is still not established (Montgelard et al. 2008; Blanga-Kanfi et al. 2009; Horn et al. 2011), continuation of the beaver examination is crucial to determine this species evolutionary origin. Searching of the available organism protein databases (Fig. 4e, f) revealed human, horse and primates as more related with beaver than species classified to mouse-related clade. Probably, it may be related with completeness of the databases for those species. Additionally, proteins may be conserved across not necessarily related species, but also with evolutionary distinct ones.

Although the placenta is an object of varied studies, most are focused on relationships with human diseases (Cox et al. 2011; Sitras et al. 2015), but rarely concern model organisms (Du et al. 2014; Saben et al. 2014). Physiological development and proper function of the placenta are under influence of sophisticated pathways related with expression of substantial genes throughout pregnancy. Therefore, any alterations affecting gene expression or posttranslational modification may lead to disorders manifested in pregnancy disorders (Majewska et al. 2017). Studies of the transcriptome provide knowledge concerning placental evolution and create the opportunity to define specific, common genes engaged in reproductive physiology in eutherians (Hou et al. 2012; Carter 2018). Previous study of the beaver revealed that the number of foetuses affects the placental transcriptome, namely 55 *C. fiber* transcripts were differentially expressed in placentas originating from twin and triple pregnancies (Lipka et al. 2017b). Different expression analysis together with present results creates general landscape of the beaver placental transcriptome. We believe that the pattern of placental expression should be established for various taxa, which can be useful for further, also interspecies studies.

Our de novo assembled placental transcriptome was functionally annotated in GO, KEGG and InterPro databases. The obtained comprehensive overview of the beaver placental transcriptome refers to placenta versatility and multi-functionality. The top represented GO terms within the biological process, molecular function and cellular component GO category were transcription, DNA-templated, binding function of ATP and integral component of membrane, respectively. In similar studies, concerning the general landscape of the human placental transcriptome (Majewska et al. 2017), overrepresented GO terms are convergent with the annotations generated in our study. Some differences in gene number assigned to each GO category may indicate interspecies variability of placenta physiology at a molecular level. However, the obtained results clearly demonstrate that despite molecular and morphological variability, the expression pattern of the human and beaver placental transcriptome is quite similar. The core set of placental expressed genes in various eutherians, overrepresented by GO terms related to ‘estrogen receptor signaling’, ‘cell motion and migration’ and ‘adherens junctions’—important for placenta functioning (Hou et al. 2012), were also identified in the beaver placenta.

Most methods to identify SNPs (single nucleotide polymorphisms) rely on the availability of a reference genome and are accurate for model species and whole genome (re-) sequencing experiments, therefore cannot be applied to non-model species (Lopez-Maestre et al. 2016). However, RNA-Seq of non-model species makes de novo assembly of SNPs computationally possible (Uricaru et al. 2015). It provides the opportunity to predict SNPs from transcribed regions that correspond to those with a more direct functional impact (Lopez-Maestre et al. 2016). In our study, parameters used for the screening process might have decreased the sensitivity in detecting rare SNVs, but reduced the chances of inclusion of false variants that arise by sequencing errors, increasing the probability of true SNV detection. Our SNV results (within CDS and UTRs) expand genetic resources for *C. fiber* that may contribute to assessing relatedness, population structure and developing conservation management based on genetic markers. Such markers are required to improve genetic selection for future reintroductions (Senn et al. 2013, 2014).

In vertebrates, more than 80% of genes include an average of 7.8–9.0 introns and > 95% of transcripts in humans are alternatively spliced, which can generate various transcripts and thus multiple protein isoforms from a single gene (Pan et al. 2008; Wang et al. 2008). It makes AS a universal regulatory mechanism which allows modulation of gene expression, phenotype and eventually causes diseases (Mourier and Jeffares 2003). AS events may modify the functional diversity in a positive or negative manner (Polo-Parada et al. 2004; An et al. 2008) and generate a tissue-specific transcriptome landscape (Wang et al. 2008). Obtained results (Fig. 2) indicate that more than 50% of highly expressed transcripts have at least two variants, in comparison to ~ 20% of transcripts from whole dataset. AS generating potentially active protein products was identified in genes known to affect significant developmental processes (Fig. 6). *ABI2* (Abl interactor 2) influence in cell–cell adhesion, cell migration and tissue morphogenesis (Grove et al. 2004). Deficiency in *Ncoa1* (nuclear receptor coactivator 1) results in embryo implantation failure and placental hypoplasia (Gehin et al. 2002; Szwarc et al. 2014). *GAB1* (GRB2-associated-binding protein 1) deficiency lead to embryonic lethality of embryos due to the heart impairment, and developmental defects of the placenta (Itoh et al. 2000). *Spint1* (serine peptidase inhibitor, Kunitz type 1) is highly expressed in the placenta (Walentin et al. 2015), and alteration in its expression cause that embryos suffer severe growth retardation or even embryonic death due to placental defects (Nagaike et al. 2008). *Abcb4* (ATP-binding cassette subfamily B member 4) as belonging to the ATP-binding (ABC) transporters may minimize the toxic effects of cadmium to the foetus by removing it out of the placenta (Liu et al. 2016). We can assume that numerous highly expressed transcripts with multiple variants (Fig. 2), as well as splice variants identified in beaver placenta (Fig. 6), may contribute to embryo development and pregnancy maintenance, as was previously described for human placenta (Soleymanlou et al. 2005; Majewska et al. 2017).

Eutherian placenta varies dramatically in size, shape, cellular composition and morphology, although its function comes to ensure an appropriate environment for a proper development of the foetuses (Furukawa et al. 2014). Differences in placental physiology between eutherians may be an adaptation to diversified reproductive strategies (Knox and Baker 2008). Our data fit into trends concerning studies of tissue-specific expression (Bogacka et al. 2017; Majewska et al. 2017; Lipka et al. 2017b). The availability of RNA-Seq data from multiple studies in the SRA database create the possibility to compare results from different tissues, physiological conditions or various species (Hou et al. 2012). To understand the evolution and biology of the placenta, further similar studies should be conducted in other species.

After a severe population bottleneck and regional extinctions through active human pressure until the early twentieth century, the re-establishment of the Eurasian beaver in Europe is considered to be a major conservation success (Frosch et al. 2014). However, more advanced reintroduction strategies and appropriate management are required to avoid a subsequent decrease in the beaver population (Halley and Rosell 2002, 2003). Exploration of the placental transcriptome enables identification of molecular pathways that are essential for the development of the placenta and ultimately determine reproductive success (Hou et al. 2012). Thus, our studies of the beaver placenta gain particular significance, due to annotations to GO categories and KEGG pathways.

Effective reproduction, especially of endangered species, is a principal requirement for conservation of biodiversity and genetic resources of different animals (McEwing et al. 2014). However, controlled reproduction may also allow a decrease in the population size of some wild animals (Kirkpatrick et al. 2011). An example of such efforts may be fertility inhibition of males (by gossypol—known as male contraceptive agent) in the deer (Gizejewski et al. 2008).

## Conclusions

Our pioneering data provide the first comprehensive view of placental transcriptome of the beaver during single and multiple pregnancy. It should serve as a valuable resource for future in-depth investigations of the *C. fiber* placental transcriptome. With respect to the functional relationship to reproduction, the identified transcripts need to be further explored to better understand important aspects of beaver physiology that contribute to reproductive performance.

## Electronic supplementary material

Table S1 Gene Ontology (GO) categorization of beaver placental genes to biological process (BP), molecular function (MF) and cellular component (CC). (XLSX 371 kb)

Table S2 Beaver placental genes classification to molecular pathways due to the Kyoto Encyclopedia of Genes and Genomes (KEGG) database. (XLSX 27 kb)
